# Atlas and Abstract of the Diseases of the Larynx

**Published:** 1899-04

**Authors:** 


Atlas and Abstract of the Diseases of the Larynx. By Dr. L. Grünwald, of Munich. Authorized translation from the German. Edited by Charles P. Grayson, M. D., Lecturer on Laryngology and Rhinology in the University of Pennsylvania; Physician in Charge of the Throat and Nose Department, Hospital of the University of Pennsylvania. With 107 colored figures on 44 plates. Published by W. B. Saunders, Philadelphia, Pennsylvania. Price, cloth, $2.50, net.

This is another of the beautiful and useful series of hand-atlasses, several of which have been previously reviewed.

I do not know of a work on the subject which will prove so useful to the general practitioner as this. The first hundred pages contains a concise but thorough treatise on the pathology, symptomatology and treatment of the diseases of the Larynx, with an excellent chapter on laryngoscopy. The remainder of the volume consists of 107 colored figures illustrating actual cases, with the clinical notes of each case on the opposite page.

The style is essentially clinical and thoroughly practical, and as before remarked, addresses itself more particularly to the general practitioner than to the specialist. The plates are beautiful specimens of art. J.

